# Most evidence for the compensation account of cognitive training is unreliable

**DOI:** 10.3758/s13421-018-0839-z

**Published:** 2018-08-16

**Authors:** Tomasz Smoleń, Jan Jastrzebski, Eduardo Estrada, Adam Chuderski

**Affiliations:** 1 Jagiellonian University, ul. Grodzka 52, 31-044 Krakow, Poland; 2 University of California, Davis, 135 Young Hall, One Shields Avenue, Davis, CA USA

**Keywords:** Training, Stimulation, Regression to the mean, Compensation effect

## Abstract

Cognitive training and brain stimulation studies have suggested that human cognition, primarily working memory and attention control processes, can be enhanced. Some authors claim that gains (i.e., post-test minus pretest scores) from such interventions are unevenly distributed among people. The magnification account (expressed by the evangelical “who has will more be given”) predicts that the largest gains will be shown by the most cognitively efficient people, who will also be most effective in exploiting interventions. In contrast, the compensation account (“who has will less be given”) predicts that such people already perform at ceiling, so interventions will yield the largest gains in the least cognitively efficient people. Evidence for this latter account comes from reported negative correlations between the pretest and the training/stimulation gain. In this paper, with the use of mathematical derivations and simulation methods, we show that such correlations are pure statistical artifacts caused by the widely known methodological error called “regression to the mean”. Unfortunately, more advanced methods, such as alternative measures, linear models, and control groups do not guarantee correct assessment of the compensation effect either. The only correct method is to use direct modeling of correlations between latent true measures and gain. As to date no training/stimulation study has correctly used this method to provide evidence in favor of the compensation account, we must conclude that most (if not all) of the evidence should be considered inconclusive.

## Introduction

In social sciences and other disciplines dealing with living organisms (e.g., medicine, agriculture), researchers often study the effects of interventions. Specifically, in cognitive and developmental psychology, recent years have brought a multitude of studies focused on the positive effects of training cognitive abilities such as working memory, attention, and reasoning. Although there is still heated debate on whether the far transfer of a trained ability, such as the increase in reasoning ability when working memory is trained, is possible (Klingberg, 2010; Jaeggi, Buschkuehl, Jonides, & Perrig, 2008) or not (Colom et al., 2013; Redick et al., 2013). There is little doubt that in terms of near transfer, the existing cognitive training methods are effective (Klingberg, 2010; Morrison & Chain, 2011; Shipstead, Redick, & Engle, 2012). More recent reports from neuroscience have even suggested the possibility of enhancing cognitive processing via non-invasive transcranial electrical stimulation with direct or alternating currents (e.g., Jaušovec & Pahor, 2017; Pahor & Jaušovec, 2014; Polanía, Nitsche, Korman, Batsikadze, & Paulus, 2012; Santarnecchi et al., 2015, 2016).

Besides the sheer effectiveness of training/stimulation for cognitive performance, a growing number of studies have investigated whether the training/stimulation gain (i.e., the difference in score between the performance recorded after training/stimulation [posttest] and the baseline performance before training/stimulation [pretest]) is distributed evenly in the trained sample (e.g., Holmes & Gathercole, 2013; Loosli, Buschkuehl, Perrig, & Jaeggi, 2012), or whether some people can be trained/stimulated more effectively than others. Two contrasting kinds of findings have been made with regard to the uneven distribution of such gains, and as a result two competing theories have been developed (see Karbach & Unger, 2014; Lövdén, Brehmer, Li, & Lindenberger, 2012).

The magnification account (gisted by the evangelical “who has will more be given”) predicts that the most cognitively efficient people at pretest will show the largest gains. Regarding cognitive training, this proposition assumes that learning to perform better on a given task, including acquisition of new skills and strategies, requires substantial involvement of cognitive resources. The more resources a person can invest in the training, the larger the gain. However, such evidence is relatively scant, and pertains primarily to teaching more effective cognitive strategies to deal with a task (e.g., Bjorklund & Douglas, 1997; Brehmer, Li, Müller, von Oertzen, & Lindenberger, 2007; Kliegl, Smith, & Baltes, 1990; Kramer & Willis, 2002; Swanson, 2014, 2015; Verhaeghen & Marcoen, 1996). Only one paper (Foster, Harrison, Draheim, Redick, & Engle, 2017) has suggested magnification effects pertaining to regular working memory training; it reported larger gains in people from the third than from the first tercile of working memory capacity after 20 sessions of either complex span or running memory task training.

In contrast to the magnification account, the compensation account of cognitive training (“who has will less be given”) predicts that the most cognitively efficient people at pretest already perform at ceiling and are not able to improve (will display negligible gains). Therefore, training will yield the largest gains in the least cognitively efficient people, who still have room for improvement, thus allowing them to catch up.

Probably the most widely discussed example of compensation account can be found in the field of intelligence (Lee et al.,, 2012, 2015; Baniqued et al.,, 2014). It has been argued that training strategies have a greater impact on performance when subjects’ baseline performance is low (Gopher, Weil, & Siegel, 1989; Espejo, Day, & Scott, 2005). Some other examples of compensation account come from children’s learning (Schneider, 2012), selective attention (Feng & Spence, 2007), executive functions (Karbach & Kray, 2016), life span development (Baltes, 1987), and from the field of expertise in which training can reduce differences between low- and high-aptitude experts (Bjorklund & Schneider, 1996). The compensation effect is also proposed as an explanation for improvements which are observed in strong decline in frontal lobe tasks (Raz, 2000).

One form of compensation proposition is the disuse hypothesis, which assumes that cognitive decline in cognitive abilities (e.g., in old age) may be caused by suboptimal use of available resources by people who have never increased their cognitive reserve (e.g., at a younger age). According to the disuse hypothesis, the decline can be reduced in groups with diminished abilities by optimizing the use of resources. However, such optimization would have a negligible effect in groups which already function at a near optimal level (Gatz et al., 2001; Ihle, Oris, Fagot, Maggiori, & Kliegel, 2016; Kliegel, Zimprich, & Rott, 2004; Sorenson, 1933).

Although, there exist studies which use brain imaging to show that cognitive training results in increased activation in regions that are less activated in a lower performing group (Hampstead, Stringer, Stilla, Giddens, & Sathian, 2012), the majority of evidence (e.g., Ball, Edwards, & Ross, 2007; Chan, Wu, Liang, & Yan, 2015; Cox, 1994; Dahlin, 2011; Gaultney, Bjorklund, & Goldstein, 1996; Karbach, Strobach, & Schubert, 2015; Kattenstroth, Kalisch, Holt, Tegenthoff, & Dinse, 2013; Willis & Nesselroade, 1990; Zinke, Zeintl, Eschen, Herzog, & Kliegel, 2012; Zinke et al., 2014) for the compensation account comes from negative correlations of baseline performance, and gains from training.

For example, in a sample of 41 children aged 9 to 12 years (Dahlin, 2011), negative correlations were observed (up to about − .5) between initial performance on the Span Board, Digit Span, Stroop, and Raven Colored Matrices tests, and a gain in performance on these tasks after five weeks of intensive working memory training using the RoboMemo task. Chan et al., (2015) trained 13 younger and 12 older adults for ten days on an adaptive *n*-back task. They pre- and post-tested them using spatial/verbal *n*-back tasks and a finger sequence learning task. The negative correlations observed between baseline performance on the latter tasks and the respective increase in performance after training was as much as *r* = −.81. Zinke et al., (2012) trained 20 older adults with five WM tasks over the course of ten sessions and observed baseline—gain correlations as strong as *r* = −.89. The other related studies cited above reported at least moderate compensation effects.

In psychology, it is barely possible to observe correlation strengths exceeding .8 (the upper limit for the strength of correlation is defined by the square root of product of the reliabilities of correlated tasks, and the reliability of psychological tests rarely exceeds .8). Thus, one has to be especially suspicious of the aforementioned evidence for the compensation account. In fact, the aim of the present paper is to demonstrate that calculating the correlations of pretest scores and gain, as is commonly adopted by proponents of this account, is a hallmark example of a statistical artifact called “regression to the mean”, dating Francis Galton (1886). By mathematical derivation (Section 2, see also Johns, 1981; Lord, 1956; Wall & Payne, 1973) and numerical simulations (Section 2), we will show that the strong negative correlations of the baseline performance and gains from training are *always* present in the data, and are driven by statistical properties of noisy repeated measurements. Thus, existing evidence is not able to support the compensation account, and the conclusions provided by virtually all studies in this vein are disputable.

However, we note that some authors are aware of the problems pertaining to the pretest-gain correlation calculations, and in order to validate the compensation effect they applied one of the two methods which should yield more correct assessment of the magnitude of this effect: either (a) an alternative variable for the gain calculation in order to avoid repeated measurements (Santarnecchi et al., 2016), (b) a formal model that includes or excludes the baseline performance × gain interaction (e.g., structural equation model, SEM, Guye, Simoni, & von Bastian, 2017; Lövdén et al., 2012), or (c) comparison of control and experimental group (e.g., Dahlin, 2011; Karbach et al., 2015; Zinke et al., 2012, 2014). Unfortunately, not all of these methods constitute an improvement compared to the (naïve) correlation of pretest and gain. In particular, using a control group is effectively of no use if the variables involved in the analysis are confounded. Only correct application of the active control group in order to investigate individual differences in training/stimulation effects may give unbiased estimation of the compensation effect. All of the aforementioned studies used the control group to compare the influence of the pretest score on the *gain* (instead of posttest) between the control and experimental groups. As we will show, the pretest and gain are related in a way that makes such an analysis faulty and only comparison of the relationship between pretest and posttest in control and experimental groups may allow biased conclusions to be avoided.

Most of the examples of studies in which the correlation between pretest and gain was used come from the cognitive training domain so we will refer to this field in this article whenever in need for an example of such a study. However on a methodological and statistical level this field is not specific in any way and we would like to underline that the problem described, solutions tested, and the final conclusions refer to every domain of empirical sciences in which the pretest—manipulation—posttest design is applied and the hypothesis on the relationship between pretest and gain is tested.

The remainder of this article has four parts. First, we show analytically that simple correlation of observed pretest and gain cannot serve as estimation of correlation of true pretest and gain. Second, we take a closer look at the strength of the observed correlation, depending on several boundary conditions. Third, we discuss possible correct methods of estimating true correlation of pretest and gain. Finally, we analyze sample data with both correct and incorrect methods in order to evaluate their accuracy.

## Analytical derivation of the persistent negative correlation between pretest and gain

In this section, we analyze the mathematical relation between one variable (e.g., pretest score) and another variable that is the result of linear combination of the first variable with a third variable (e.g., the difference between post-test and pretest scores). This kind of statistical model is often used to detect a nonlinear relationship between two variables. For example, in line with the compensation account, one can predict that people with lower cognitive ability level will benefit to a greater extent from cognitive training than people with a higher ability level (who already perform optimally). One can then correlate the pretest ability test score with the difference between post-test and pretest (i.e., gain) and interpret the negative correlation that is usually observed in such a case as a direct confirmation of the compensation hypothesis. It will be demonstrated that such a method may not be the best idea. Put simply, correlation statistics calculated in this way do not provide reliable estimation of the true correlation between pretest and gain.

In the present argument, we make only one assumption: that observed measures constitute the sums of some true, unobserved values and random independent noise. This is definitely a very weak assumption in light of the fact that most psychological studies do not directly tap the constructs measured, but rely on tools (e.g., tests, questionnaires, etc.) that show only imperfect reliability.

Let us consider two observed variables, *O*_1_ and *O*_2_. Each is the sum of some true (unobserved) value (*V*_1_ and *V*_2_, respectively) and random noise (*ε*_1_ and *ε*_2_, respectively). Of interest is the relationship between true value *V*_1_ and the difference Δ between true values *V*_2_ and *V*_1_. Since one cannot directly access these true values, the relationship in question must be estimated by using the observed values of variables (i.e. *O*_1_ and *D* = *O*_2_ − *O*_1_).

Our argument can easily be generalized onto any linear relation between variables *O*_1_ and *O*_2_ defined as above (which obviously does not need to come from pretests and posttest), but for the sake of simplicity we henceforth focus on a convenient (and commonly reported in cognitive training studies) example relation pertaining to the difference between the two variables (gain).

The correlation of *O*_1_ and *D* is a biased estimator of the correlation of *V*_1_ and Δ. Specifically, the difference *D* between *O*_2_ and *O*_1_ equals Δ + *ε*_1_ + *ε*_2_:

$$\begin{array}{@{}rcl@{}} D & =& O_{2} - O_{1}\\ & =& V_{2} + \varepsilon_{2} - (V_{1} + \varepsilon_{1})\\ & =& V_{2} + \varepsilon_{2} - V_{1} + \varepsilon_{1}\\ & =& V_{2} - V_{1} + \varepsilon_{1} + \varepsilon_{2}\\ & =& {\Delta} + \varepsilon_{1} + \varepsilon_{2}. \end{array} $$

Note that − (*V*_1_ + *ε*_1_) equals − *V*_1_ + *ε*_1_ because *ε*_1_ is independent random noise. Adding such a noise to any variable has the same effect as subtracting it from that variable.

Now, why is it a bad idea to estimate the correlation of *V*_1_ and Δ (true pretest and gain) by assessing the correlation of *V*_1_ + *ε*_1_ and Δ + *ε*_1_ + *ε*_2_ (observed pretest and gain)? The Pearson product-moment correlation coefficient *r*_*X*,*Y*_ of *X* and *Y* equals the covariance of *X* and *Y* (cov(*X*,*Y* )) scaled by the product of the standard deviations of these variables (*σ*_*X*_*σ*_*Y*_):

1 $$ r_{X,Y}= \frac{\operatorname{cov}(X,Y)}{\sigma_{X} \sigma_{Y}}. $$

As the scales are irrelevant here, the means of *X* and *Y* can be fixed to 0 and the product of their standard deviations (*SD* s) can be fixed to 1. In which case the denominator also equals 1. Consequently, the strength of correlation between the variables equals the covariance between them.

The covariance between *X* and *Y* is an expected value of the product of differences between each variable and its mean:

$$\operatorname{cov}(X,Y) = \operatorname{E}\left( (X-\mu_{X})(Y-\mu_{Y})\right). $$

Because the means of *X* and *Y* both equal 0 (due to our choice of scale), the formula can be simplified to:

$$\operatorname{cov}(X,Y) = \operatorname{E}(XY). $$

As, by assumption, both *X* and *Y* are the sums of variables (*X* = *A* + *α* and *Y* = *B* + *β*), the latter formula can be rewritten in the following way:

$$\operatorname{cov}(X,Y) = \operatorname{E}\left( (A + \alpha) (B + \beta)\right), $$

and rearranged to:

$$\operatorname{cov}(X,Y) = \operatorname{E}(A B + A \beta + B \alpha + \alpha \beta), $$

which, due to the feature of linearity of expected value equals:

2 $$ \operatorname{cov}(X,Y) = \operatorname{E}(A B) + \operatorname{E}(A \beta) + \operatorname{E}(B \alpha) + \operatorname{E}(\alpha \beta). $$

Now, let us interpret both *X* and *Y* as observed measures, both *A* and *B* as the true unobserved value of some psychological variable, and finally both *α* and *β* as independent random noise. In consequence, certain corollaries can be derived from all the previous assumptions. First, from the zero mean of both *X* and *Y* (because of our choice of scale), and from the zero mean of both *α* and *β* (the intrinsic feature of noise), there follows the zero mean of both *A* and *B*. The latter fact implies that the expected value of the product of any pair of variables in the formula (2) equals the covariance of these two variables,

$$\operatorname{cov}(X,Y) = \operatorname{cov}(A,B) + \operatorname{cov}(A,\beta) + \operatorname{cov}(B,\alpha) + \operatorname{cov}(\alpha,\beta). $$

When two variables are independent then their covariance equals 0. Thus, both cov(*A*,*β*) and cov(*B*,*α*) yield zero because the random noise (e.g., *β*) of one measure (e.g., *Y* ) cannot be related to the true value of another variable (e.g., *A*). Consequently:

$$\operatorname{cov}(X,Y) = \operatorname{cov}(A,B) + \operatorname{cov}(\alpha,\beta), $$

therefore, the covariance of *X* and *Y* equals the covariance of *A* and *B* if and only if the covariance of *α* and *β* equals 0. From the above, it follows that if *α* and *β* are dependent, then cov(*X*,*Y* ) constitutes a biased estimator of cov(*A*,*B*).

Consequently, when one is computing the correlation between observed variable *O*_1_ and the difference *D* between *O*_1_ and another observed variable *O*_2_, in fact one is computing the correlation between *V*_1_ + *ε*_1_ and Δ + *ε*_1_ + *ε*_2_, even though one is aiming to estimate the correlation between true value *V*_1_ and the difference Δ between true values *V*_2_ and *V*_1_. Correlation strength computed in this way will be different than the true strength of the relationship in question because random terms *ε*_1_ and *ε*_1_ + *ε*_2_ are clearly not independent. As a result, simply because of the statistical properties of the measures used, a researcher will likely observe an incorrect correlation strength between a given pretest score and a relative gain in a post-test score, compared to the true strength (if any) of the relationship between the pretest score and the gain.

## Analysis of several specific cases of incorrect pretest-gain correlation

The strength of correlation between pretest and gain was analyzed in several specific cases in order to assess the magnitude of difference between the true versus the observed correlation of these variables.

### How large is the difference between true and observed pretest-gain correlation?

First, the relationship between pretest and gain was examined in a case in which there is no relation between pretest and posttest and their variance is equal.

Let us remind the reader that gain (*D*) is the difference between posttest (*O*_2_) and pretest (*O*_1_). Variance (*σ*^2^) of the difference between variables is the sum of variances of the variables, decreased by the covariance between them multiplied by two (feature of covariance):

$${\sigma^{2}_{D}} = \sigma^{2}_{O_{1}} + \sigma^{2}_{O_{2}} - 2 \operatorname{cov}(O_{1},O_{2}). $$

Knowing ${\sigma ^{2}_{D}}$, one can compute the covariance between pretest and gain (feature of covariance):

3 $$ \operatorname{cov}(O_{1},D) = \frac{\sigma^{2}_{O_{2}} - \sigma^{2}_{O_{1}} - {\sigma^{2}_{D}}}{2}, $$

then, by putting the formula (3) into the formula (1) one can compute the correlation between the variables. Accordingly, when the variances of both pretest and post-test are equal and there is zero correlation between them, the expected value of correlation between pretest and gain equals − .71.

### What determines the size of the difference between true versus observed pretest-gain correlation?

**Fig. 1:**
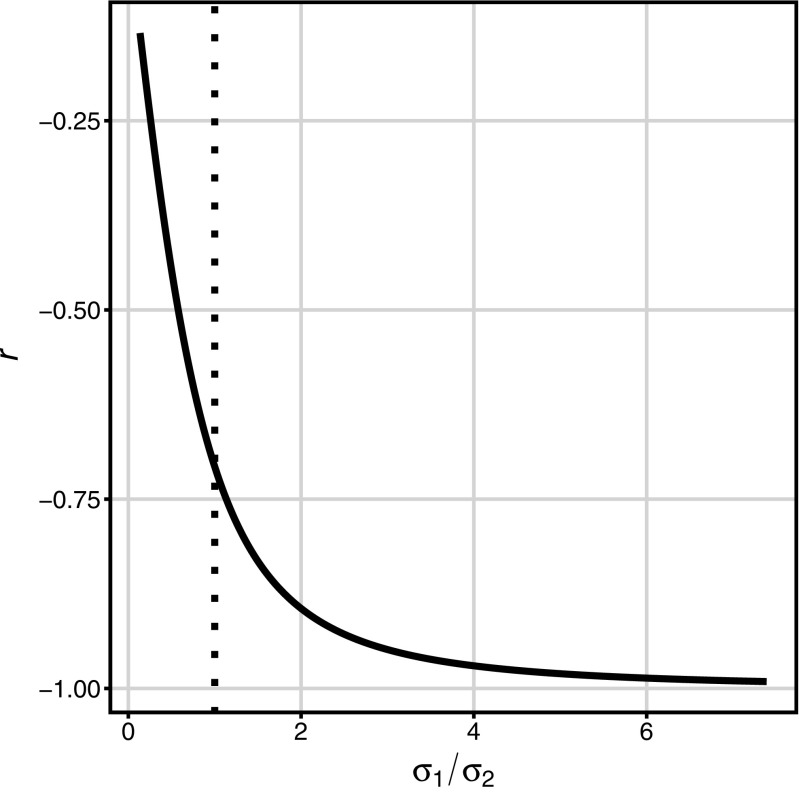
Relation between the ratio of pretest standard deviation (*σ*_1_) to post-test standard deviation (*σ*_2_), and the observed correlation between pretest and gain (*r*, *solid line*). The *dotted line* marks point of equal standard deviations of pretest and post-test indicating *r* = −.71

As the correlation of two variables can be affected by a change in the variance of one variable, given that the other variable’s variance and both variables’ means are fixed, next it was examined how the difference between true versus observed correlation varied depending on the relative disparity in variance between pretest and post-test. Figure 1 shows a decreasing hyperbolic relationship between pretest variance (varying from 0.14 to 7.14, while the post-test variance equaled 1) and the correlation between pretest and gain. This very function implies that with an increasing range of pretest values, the size of the difference between the true versus the observed pretest-gain correlation will increase, approaching *r* = − 1 (i.e., the perfect negative correlation, while the true correlation is null) when the pretest *SD* becomes at least five times larger than the posttest *SD*.

### What is the size of the difference between true versus observed pretest-gain correlation when there actually is a relationship between pretest and gain?

Let us assume that post-test is a linear function of pretest, given by the formula *V*_2_ = *βV*_1_ + *ζ* (where *β* is change due to intervention and *ζ* is random noise). When *β* equals 1, there is no relationship between the pretest and the gain (the change due to intervention does not depend on base performance). For values larger than 1, there is a positive relationship (predicted by the magnification account). For values smaller than 1, there is negative relationship (predicted by the compensation account).

In order to examine example cases of the relationship between pretest and gain, we analyzed how the correlation between these variables depends on the relationship between pretest and post-test (*β*), the variance of prediction error of pretest and post-test (*ζ*), and the variance of measurement error of pretest and post-test (*ε*, since *O*_2_ = *V*_2_ + *ε*).

The correlation (*ρ*) between true pretest *V*_1_ and true gain Δ can be obtained from the covariance of these variables and their standard deviations according to formula (1). Next, the covariance can be computed from the formula (feature of covariance):

4 $$ \operatorname{cov}(V_{1},{\Delta}) = \frac{\sigma^{2}_{V_{2}} - \sigma^{2}_{V_{1}} - \sigma^{2}_{{\Delta}}}{2}. $$

The true pretest variance ($\sigma ^{2}_{V_{1}}$) depends on the choice of scale and can be set to 1. The true post-test variance ($\sigma ^{2}_{V_{2}}$) equals $\beta ^{2} \sigma ^{2}_{V_{1}} + \sigma ^{2}_{\zeta }$ (which follows from the definition of *V*_2_). The true gain variance ($\sigma ^{2}_{{\Delta }}$) equals $\sigma ^{2}_{V_{1}} + \sigma ^{2}_{V_{2}} - 2 \beta \sigma ^{2}_{V_{1}}$ (obvious proof of the latter claim lies beyond the scope of this article).

Analogously, we can infer the correlation (*r*) between the observed pretest (*O*_1_) and the observed gain (*D*). The observed pretest variance ($\sigma ^{2}_{O_{1}}$) equals $\sigma ^{2}_{V_{1}} + \sigma ^{2}_{\varepsilon }$, the true pretest variance ($\sigma ^{2}_{O_{2}}$) equals $\sigma ^{2}_{O_{2}} + \sigma ^{2}_{\varepsilon }$, and the observed gain variance (${\sigma ^{2}_{D}}$) equals $\sigma ^{2}_{O_{1}} + \sigma ^{2}_{O_{2}} - 2 \beta \sigma ^{2}_{V_{1}}$.

**Fig. 2:**
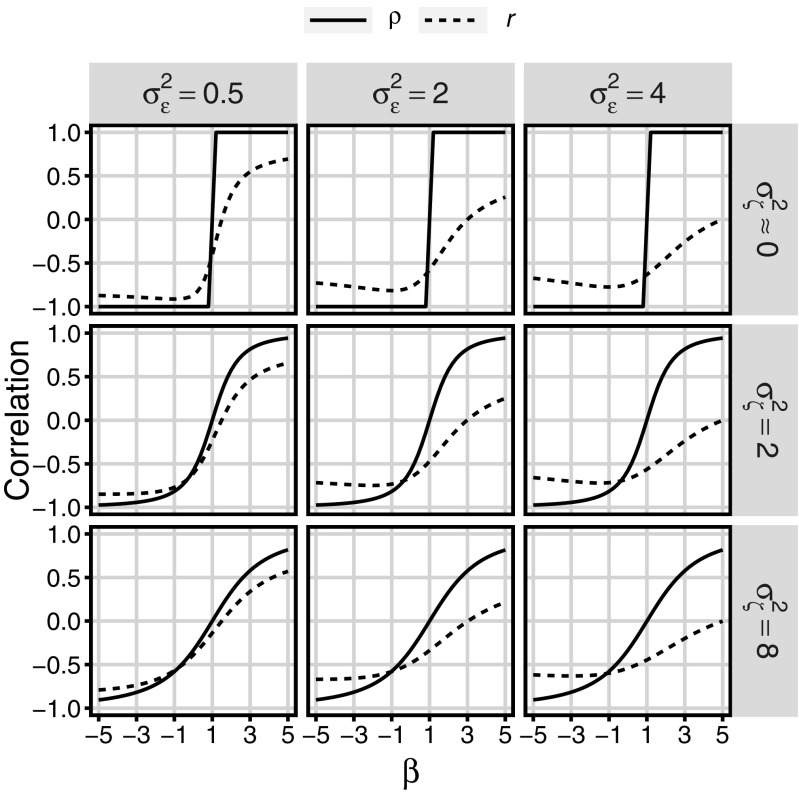
True correlations (*ρ*) between true values (*V*_1_ and Δ) and observed correlations (*r*) of observed values (*O*_1_ and *D*), as a function of *V*_2_ regression coefficient on *V*_1_ (*β*), the variance (*σ*^2^) in prediction error (*ζ*) and the variance in measurement error (*ε*). *β* smaller than 1 expresses compensation effect; *β* greater than to 1, magnification effect; *β* equal to 1, lack of either of the effects. When variance in prediction error is null the true correlations is perfectly negative (for compensation) or perfectly positive (for magnification). The larger the variance in prediction error the weaker the correlation (both true and observed) for both these effects. When variance in measurement error is null, *r* is equal to *ρ* for all *β* (not shown on the plot). The larger the variance in measurement error the more underestimated are both compensation and magnification effects

Figure 2 shows the relationship between the true and observed correlation of pretest and gain, the regression slope, and both types of error (*ζ* and *ε*). True correlation (*ρ*) was computed according to the formula (1), whereas covariance was provided on the basis of the formula (4). Analogously the observed correlation (*r*) was computed on the basis of the formulas (1) and (3). It can be noticed that the higher the variance of measurement error, the larger the discrepancy between true and observed correlation. Also, the higher the variance of prediction error, the smaller the strength of both true and observed correlation for all values of *β*.

## Evaluation of alternative methods of analysis of the relationship between pretest and gain

Apart from the naïve correlation between pretest and gain, there are three quite straightforward statistical methods that can be used to examine the relationship between one variable and another variable that is a linear function of the former. In the remainder of the paper, each such method will be tested against artificially generated data (with and without the compensation effect).

The mathematically simplest method is to compute the correlation in question between the gain and another independent measure of the true pretest score (e.g., Santarnecchi et al.,, 2016). The noise in both variables will be unrelated, but the method requires additional data (another measure). The second method is to test the relative fit to data of regression models that include either unit relationship (slope coefficient) between pretest and post-test (no relationship between pretest and gain), or relationship of magnitude other than 1 (positive or negative relationship between pretest and gain). The mathematically most complex method is to define the pretest, the gain, and their relationship with either a graphical or a structural equation model (e.g., Lövdén et al., 2012).

Each method requires only data from the experimental (i.e., either trained or stimulated) group. Additionally, a researcher may introduce the control group, and then simply compare the compensation effects in both groups. If the experimental group yields a significantly stronger pretest × gain negative (underadditive) interaction than the interaction that would (naturally) occur in the control group, then the compensation effect due to intervention may be validly argued for. Unfortunately, the control group requires planning ahead, which is a substantial change to the study’s design (e.g., doubling the sample size, defining the active control procedure, providing that the groups differ only in this procedure, etc.). The following analyses aim to determine if the control group actually is or is not necessary for any valid inferences pertaining to the compensation effect.

### Correlation with another measure

The first method is to simply use a different measure of performance in the post-test. Such an alternative measure will not share the measurement error with the gain based on the original variable (or vice versa), so the resulting observed correlation will not be a biased estimation of the true correlation between pretest and gain.

### Using regression

Alternatively, and especially when no parallel scores are available, a linear regression model can be used. The hypothesis that the gain is not related to pretest can be expressed in another way: the coefficient of regression of post-test over pretest equals 1. More specifically, the hypothesis that a higher pretest value will yield a higher gain value is equivalent to the hypothesis that the slope of the regression line of the post-test over the pretest is higher than 1. On the other hand, the hypothesis that a higher pretest value will yield a lower gain value is equivalent to the hypothesis that the slope of the regression line of the post-test over the pretest is lower than 1.

For example, if a hypothesis states that the higher pretest value (*O*_1_), the higher the gain value (*O*_2_ − *O*_1_), one can fit two linear models, *M*_1_: *O*_2_ = *α* + *O*_1_ + *ε* and *M*_2_: *O*_2_ = *α* + *βO*_1_ + *ε* (with possible restriction: *β* > 1), and can compare them with a model comparison tool (e.g., ANOVA). If model *M*_2_ proves a significantly better fit to data than model *M*_1_, then the hypothesis about the positive relation can be considered corroborated.

It should be mentioned that actually one is not allowed to use linear regression when independent variables contain noise because in such a case linear regression’s assumption of weak exogeneity is undermined (Chesher, 1991). But let us acknowledge the elephant in the room: this assumptions is hardly ever true. Most psychological studies measure independent variables with error, and in practice this does not preclude using linear regression and similar tools in statistical analysis.

### Using graphical and structural equation models

The third solution requires using a more powerful analytical framework that allows for the direct modelling of relationships between observed (manifest) and theoretical (latent) variables. Two tools commonly used for this purpose are graphical models (Koller & Friedman, 2009) and structural equation models (SEM, Kline, 2016). Each avoids the pitfalls resulting from the interaction of pretest noise and gain noise because they allow the relationship between the true (noise free) variances of pretest and post-test to be modeled. Both graphical models and SEM can be defined in many ways, so there is probably no established form of model for the problem discussed here (for examples see Section 2). Although graphical/structural equation models are more powerful than regression models, they usually incur the cost of using larger samples and multiple manifest measures.

## Analysis of the validity of compensation effect detection in simulated data

A series of simulations was performed in order to establish which (if any) of the methods described above yields an acceptable estimate of the compensation effect (when the effect is present in the data), or which can validly detect the lack of the effect (when it is absent). 10,000 simulations were run in order to achieve a level of accuracy higher than 99%, taking into account the established variance of the parameters of interest, the effect size and *α* value of .05. (Burton, Altman, Royston, & Holder, 2006). We used 5% trimmed means of parameters estimated in simulated data because, unlike the untrimmed mean, it is robust to a moderate number of outliers. The measures of error were estimated in the same way as parameters of interest (mean value of measures in sampled datasets). This method gave estimates identical to the ones based on measures of standard error calculated as the standard deviations of the simulated parameters of interest (see Schafer & Graham, 2002).

Values of *p* were not computed directly as trimmed means of simulated samples because when *p* is close to zero (e.g., when there is significant or near significant test result), the distribution of this statistic tends to be visibly skewed. Such a skewed distribution mean is vulnerable to possible outliers which are results of sampling errors. Instead, we sampled statistics whose distributions were more stable (e.g., *t* or *χ*^2^) and computed *p* values based on trimmed means of these statistics and respective degrees of freedom.

The sampling error, estimation of uncertainty, and significance of the effects depends on the sample size. So, in order to cover a wide range of possible expressions of the compensation effect, we introduced several sample sizes in the simulations. These sample sizes were chosen in order to reflect the ones used in the studies which we referenced as examples of questionable support for the compensation effect. We tested the validity of the statistical methods in small (*N* = 28 similarly as used by Karbach et al.,, 2015), medium (*N* = 48 similarly as used by Chan et al.,, 2015), large (*N* = 80 similarly as used by Zinke et al.,, 2014), and huge (*N* = 300 as a kind of best case scenario) samples. Note that due to the differences in the methodology and the statistical methods used, the *N* values are merely based on sample sizes used in the cited studies rather than mirror them.

The first dataset contained a real compensation effect, i.e., the true unobserved gain was forced to negatively correlate with the true pretest value. In the second dataset, the false compensation effect could only have appeared as a result of regression to the mean as the true gain was uncorrelated with the pretest. The third dataset used in the analysis including a control group was composed of two sets of data in equal proportions. The first half (imitating the experimental group) was generated identically to first dataset (which included covariance between pretest and gain) while the second half (imitating the control group) was generated identically to the second dataset (contain no covariance between pretest and gain).

### Artificially generated datasets

A sample of *N* data points (28, 48, 80, or 300), each defined in two dimensions (pretest and gain), was drawn from the bivariate normal distribution with the following covariance matrix

$$\left[\begin{array}{cc} 1 & -0.4 \\ -0.4 & 0.5 \end{array}\right]. $$

The first vector, generated with a mean of 0 and a variance of 1, represented the values of the true unobserved pretest (*V*_1_). The second vector, with a mean of 0.8 and a variance of 0.5, reflected the values of the true unobserved gain (Δ). The pretest and gain were negatively correlated at *r* = −.57. The mean of *V*_1_ (0) is an average pretest value; a mean of Δ (0.8) is an average value of increase in post-test compared to pretest.

Next, the values of the true unobserved post-test were computed as the sum of the two variables (*V*_2_ = *V*_1_ + Δ). Finally, the observed values of both pretest (*O*_1_ = *V*_1_ + *ε*_1_) and posttest (*O*_2_ = *V*_2_ + *ε*_2_) were calculated, where *ε*_1_ and *ε*_2_ were random noise drawn from the normal distribution (*N* = 1000,*μ* = 0,*σ* = 0.7). In a similar way, the alternative observed measures of the pretest (*O*1′ = *V*_1_ + *ε*_3_) and post-test (*O*2′ = *V*_2_ + *ε*_4_) were computed.

The second dataset was generated in the same way as the first, with the sole difference that there was no correlation between the true pretest and the true gain. The covariance matrix defining this dataset was as follows

$$\left[\begin{array}{cc} 1 & 0 \\ 0 & 0.5 \end{array}\right], $$

thus, there was no real compensation effect in the data.

The third dataset consisted of two subsets of equal sizes generated identically to the first and second datasets. Each subset was respectively labeled as “experimental” or “control” group. The sizes of the entire dataset was the same as sizes of the first and second datasets (28, 48, 80, or 300).

### Analysis of compensation effect detection

#### Naïve correlation of pretest and gain

The correlation of pretest and gain was significant and negative in all cases except the second dataset (spurious compensation) for *N* = 28; this means the method incorrectly signaled as significant a correlation that was actually null in the cases of medium, large, and huge samples (see Table 1). The correlation in the first dataset (all sample sizes) did not differ significantly from the true correlation (− 0.57) and the estimation was only slightly magnified.

**Table 1:** Faulty estimation of relationships between pretest and gain, with simple correlation

Dataset	1 (real compensation)			2 (regression to the mean)		
	*r*	95%CI	*p*	*r*	95%CI	*p*
*N* = 28	− .59	[−.79,−.29]	< .001 ***	− .33	[−.62,.04]	.076
*N* = 48	− .6	[−.75,−.38]	< .001 ***	− .33	[−.56,−.06]	.018 *
*N* = 80	− .6	[−.72,−.44]	< .001 ***	− .33	[−.51,−.12]	.0026 *
*N* = 300	− .6	[−.67,−.52]	< .001 ***	− .33	[−.43,−.22]	< .001 ***

#### Correlation of gain with alternative pretest measure

In contrast to the naïve correlation computed in the first step, the correlation between the gain and the alternative measure of pretest performance correctly detected the lack of a significant relationship between these two variables for all sample sizes. However, the method did not detect the current relationship in small and medium sample of the first dataset. In the large and huge samples, for which the correlation was correctly identified, its strength was underestimated (see Table 2).

**Table 2:** Correlation of gain with alternative pretest measure

Dataset	1 (real compensation)			2 (regression to the mean)		
	*r*	95%CI	*p*	*r*	95%CI	*p*
*N* = 28	− .26	[−.57,.11]	.15	0	[−.36,.36]	.998
*N* = 48	− .27	[−.51,.01]	.06	0	[−.28,.28]	.993
*N* = 80	− .27	[−.46,−.053]	.015 *	0	[−.22,.22]	> .999
*N* = 300	− .27	[−.37,−.16]	< .001 ***	0	[−.11,.11]	> .999

* *p* < .05, ** *p* < .01, *** *p* < .001

#### Linear regression model

The model Posttest = *α* + *β* Pretest + *ε* was fit to all the generated datasets. The estimated Shapiro–Wilk tests results revealed that the residuals were normally distributed in all models (the smallest *W* = .96, *p* = .5, observed for no compensation dataset, *N* = 28) and the models were homoscedastic (as revealed by the estimations of Breusch—Pagan test results, the largest *BP*[1] = 0.82, *p* = .49, observed for real compensation dataset, *N* = 28).

For the first dataset, in which the compensation effect was present, the linear models provided slope parameters significantly smaller than one, correctly indicating the existence of the effect. However, for the second dataset, in which the compensation effect was absent, the models provided the correct result (the slope was not significantly different from 1) only for the small sample and falsely signaled compensation (*B* significantly smaller than one) for the medium, large, and huge samples (see Table 3).

**Table 3:** Estimation of pretest-posttest slope with linear regression model

Dataset	1 (real compensation)			2 (regression to the mean)		
	*B*	95%CI	*p*	*B*	95%CI	*p*
*N* = 28	0.4	[0.08, 0.72]	.001 **	0.67	[0.29, 1.05]	.088
*N* = 48	0.4	[0.17, 0.64]	< .001 ***	0.67	[0.39, 0.95]	.022 *
*N* = 80	0.4	[0.22, 0.58]	< .001 ***	0.67	[0.46, 0.88]	.0028 **
*N* = 300	0.4	[0.32, 0.49]	< .001 ***	0.67	[0.57, 0.77]	< .001 ***

*Note:* Value of *p* is computed in reference to *B* = 1 as a null hypothesis

* *p* < .05, ** *p* < .01, *** *p* < .001

#### Structural equation models

Two alternative structural equation models were fitted to each dataset. The models were based on Lövdén et al., (2012), who tackled a similar problem of estimating the correlation between some baseline performance and a subsequent gain. The models belong to the latent difference score class; this means that the difference (gain) between the observed variables (post-test and pretest) is represented as a latent variable, and one of the observed variables (e.g., post-test) is the sum of another observed variable (e.g., pretest) and the latent gain (McArdle, 2009). This approach lets us directly model all parameters of the difference (i.e., mean, variance, covariance of the pretest with the change, etc.). Also, in the latent difference score model we can examine the statistical properties of the change without actually calculating the change scores. Latent difference score modeling is also fit for our purposes because it rests on assumptions similar to the ones made in this article (see McArdle & Hamagami, 2001).

**Fig. 3:**
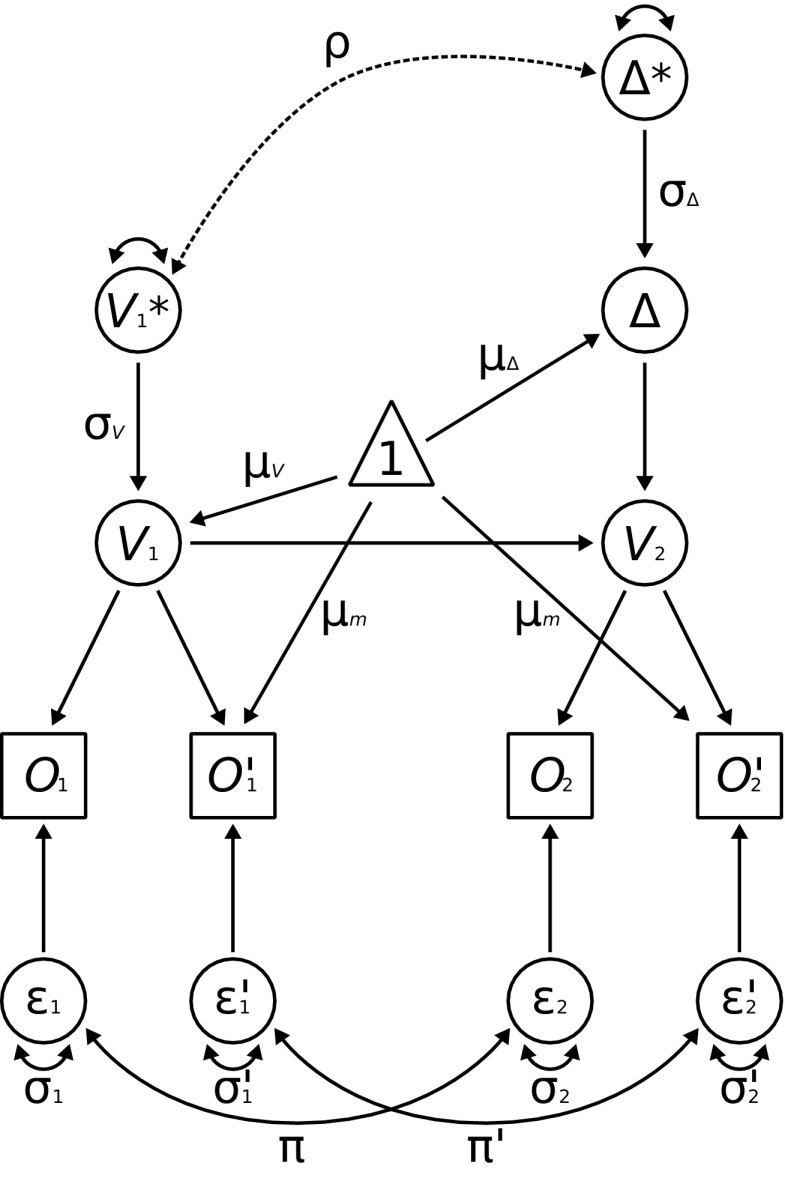
Structural equation models (based on Lövdén et al., 2012). *Squares* represent manifest variables (*O*_1_ is the score in the observed pretest, $O_{1}^{\prime }$ is the score in alternative measures of the observed pretest, and *O*_2_ and $O_{2}^{\prime }$ are scores in post-tests). *Circles* represent latent variables, *V*_1_ reflects unobserved pretest performance, *V*_2_ reflects unobserved post-test performance, and Δ indicates gain. The *triangle* represents a constant. Each *solid arrow* represents a nonzero parameter. Each named *arrow* represents a free parameter. The models differ only in the *ρ* parameter (*dashed arrow*), which represents the correlation between variance of baseline performance (*V*_1_ ∗) and variance of gain (Δ ∗). In the first model *ρ* is fixed to 0, whereas in the second model this parameter is free

Both models consisted of four manifest variables which were scores of four observed measures (see Fig. 3), *O*_1_, *O*_2_, *O*1′, and *O*2′, the pretest and the post-test of the primary, and the pretest and the post-test of the alternative measure, respectively. The residual terms of each pair of manifest measures (i.e., primary and alternative) were allowed to correlate. Both pretest scores were loaded by the latent variable representing the unobserved pretest performance (*V*_1_), and both post-test scores were loaded by the unobserved post-test latent variable (*V*_2_). The post-test variable was the sum of the pretest variable and another latent variable reflecting gain (Δ). As both *V*_1_ and Δ were linked to the constant term and variance of *V*_1_ ∗, and Δ ∗ was fixed to 1, their regression loadings on *V*_1_ and Δ equaled the standard deviation of the variables and therefore the covariance between *V*_2_ ∗ and Δ ∗ equaled the correlation between them. Two additional variables for *V*_1_ (*V*_1_ ∗) and Δ (Δ ∗, with variance fixed to 1 in both) are specified only to have a direct estimation of the correlation between them. If we correlated the variables directly, the models would be mathematically equivalent to the tested ones, but we would have a covariance estimated instead of a correlation. Also, the mean of the alternative measure was fixed to the constant term. The sole difference between the two models was that the first did not allow for the correlation between variance terms (*V*_2_ ∗ and Δ ∗), whereas the second did include such a correlation (Fig. 3, dashed line).

It must be stressed here that fitting SEM for most of sample sizes used in this simulation is generally a very precarious idea and should be done only in cases when it can be demonstrated that the small sample size does not influence the reliability of the results (Kline, 2016). In case of described simulations, the relatively small sample sizes are not problematic because (a) the large number of simulation removes problem of sampling error, (b) artificial generating of the data provides that tested models are true and (c) that all sources of error are known and controlled.

We fitted the models with Lavaan (0.5) using unstandardized input and maximum likelihood estimation. In the first dataset (real compensation), only the first model (which included pretest-gain correlation) achieved a satisfactory fit for all sample sizes. The second model (which assumed no correlation of pretest and gain) failed to meet the criteria of acceptability for any sample size. Additionally, the parameter of the correlation between pretest and gain (*ρ*) in the first model was significantly negative for all sample sizes. In the second dataset (no real compensation effect), both models achieved satisfactory fit. The measures of fit were very similar in both models, with the second model displaying a moderate advantage (*χ*^2^ and *CFI* were either inconclusive or slightly favored the first model; *RMSEA* was either inconclusive or slightly favored the second model; *AIC* and *BIC* slightly favored the second model). More explicit evidence in favor of the second model was provided by estimations of *ρ*. For all sample sizes, the parameter did not differ significantly from zero; in fact, the estimated value of the parameter was very close to zero. Full comparison of the fit measures is demonstrated in Table 7. Table 4 presents brief comparison based on the relative *χ*^2^. This measure is used to test the hypothesis about the increase in model’s fit as free parameters are added to the model. The model which has more free parameters will always have better fit than the model with lower number of free parameters because it is more complex/flexible than the latter one but the increase in fit does not always compensates the increase in the complexity. The relative *χ*^2^ can be used to compare nested models fitted to the same dataset taking into account both the models’ fit and complexity. The significant difference means that the more complex model is the better one.

**Table 4:** Brief of the comparison of the goodness-of-fit measures for the two structural equation models

Dataset	1 (real compensation)				2 (regression to the mean)			
	Relative *χ*^2^	*p*	*ρ*	95%CI	Relative *χ*^2^	*p*	*ρ*	95%CI
*N* = 28	4.05	.044 *	− .58	[− 1,−.074]	0.89	.34	.057	[−.72,.83]
*N* = 48	6.33	.012 **	− .57	[−.9,−.22]	0.85	.36	.034	[−.52,.57]
*N* = 80	10	.0016 **	− .57	[−.82,−.31]	1.04	.37	.018	[−.39,.42]
*N* = 300	36.17	< .001 ***	− .57	[−.69,−.44]	0.81	.37	0.0045	[−.19,.2]

*Note:* *DF* for relative *χ*^2^ equals 1

For complete comparison see Table 7 in Appendix

* *p* < .05, ** *p* < .01, *** *p* < .001

#### Using control group

The linear regression model including the interaction between group and pretest was fitted to the third dataset, which contained two halves (first, including covariance between pretest and gain—experimental group; and second, which did not include such a correlation—control group). The model was defined as follows: Posttest = *α* + *β* Group + *γ* Pretest + *δ* Group×pretest + *ε*. The estimated Shapiro—Wilk test results revealed that the residuals were normally distributed in all models (the smallest *W* = .96, *p* = .5, observed for *N* = 28) and the models were homoscedastic (as revealed by the estimations of Breusch—Pagan test results, the largest *BP*[1] = 6.37, *p* = .19, observed for *N* = 300).

The resulting model parameters are presented in Table 8 in Appendix. Comparison of estimations of the interaction parameter is presented in Table 5. The interaction in question was significantly different from zero only for huge sample size; this means that the linear model failed to detect that the experimental group displayed a larger compensation effect than the control group for all “realistic” sample sizes. This fact led to the invalid conclusion that in the experimental group the compensation effect did not consist of anything more than the artifactual compensation effect (as in the control group).

**Table 5:** Estimation of interaction parameter (group × pretest) in linear regression model (dataset 3)

	*B*	95%CI	*p*
*N* = 28	− 0.27	[− 1.02, 0.48]	.45
*N* = 48	− 0.27	[− 0.8, 0.27]	.31
*N* = 80	− 0.27	[− 0.67, 0.14]	.19
*N* = 300	− 0.27	[− 0.47,− 0.067]	.0088 **

For estimation of all parameters in the model see Table 8 in Appendix

* *p* < .05, ** *p* < .01, *** *p* < .001

### Conclusions

Three patterns can be observed among the results of the application of the analysed statistical methods (see Table 6). First, both correlation with alternative measure and analysis of interaction using the control group seem to be able to correctly diagnose the presence and absence of the compensation effect, provided they have enough power. In the analyzed datasets, the correlation with alternative measure failed to detect the existent compensation in small and medium samples; however, the method almost indicated the effect in the medium sample (*p* = .06) and correctly detected the effect in the large and huge samples. Similarly, analysis of interaction using the control group revealed a negative interaction between group and pretest value only in huge sample, therefore the hypothesis about a higher compensation effect in the experimental group (both real and artifactual compensation) than in the control group (sole artifactual compensation) was not confirmed for all sample sizes based on real studies. However, the estimate of the interaction was invariably negative for all sample sizes and it was the large standard error that made the effect insignificant. Nevertheless, the error systematically decreased with the increase of sample size and ultimately the interaction reached significance for the huge sample. Therefore, one can undoubtedly expect that if sufficient power is provided (low measurement error, large sample, etc.) this method will correctly detect an existing compensation effect.

**Table 6:** Diagnosis of compensation effect by all tested statistical methods

Dataset	1 (real compensation)				2 (regression to the mean)				3 (combined dataset)
	NC	CWAM	LM	SEM	NC	CWAM	LM	SEM	LMWI
*N* = 28	**detected**	rejected	**detected**	**detected**	**rejected**	**rejected**	**rejected**	**rejected**	not discriminated
*N* = 48	**detected**	rejected	**detected**	**detected**	detected	**rejected**	detected	**rejected**	not discriminated
*N* = 80	**detected**	**detected**	**detected**	**detected**	detected	**rejected**	detected	**rejected**	not discriminated
*N* = 300	**detected**	**detected**	**detected**	**detected**	detected	**rejected**	detected	**rejected**	**discriminated**

*Note:* Correct outcomes are printed in bold. NC—Naïve correlation, CWAM – Correlation with alternative measure, LM—Linear model, LMWI—Linear model with interaction

Second, both the naïve correlation and the linear model appeared to be quite unreliable. On one hand, naïve correlation provided the correct diagnosis for small and medium samples and the linear model provided correct diagnosis for the small sample, but it is clear that neither method falsely diagnosed the inexistent compensation effect for the small (or medium) sample size simply due to the large standard error of the estimation, which was consequence of the sample size. Thus, when using these methods, one finds oneself in an awkward situation where the higher the power of the test, the higher the chance of obtaining the wrong outcome.

Finally, structural equation models proved to be an accurate tool for the task. For all sample sizes of the first dataset, SEM unequivocally indicated the first of the models (which assumed correlation between pretest and gain) as better than the alternative one. Also, estimation of the correlation parameter was definitely negative. So, the conclusion about the existence of the compensation effect was consistently true. In the second dataset, SEM indicated the second model (which assumed no correlation between pretest and gain) as better. However, the fit measures were far less unequivocal than in the first dataset. Some of the fit measures indicated the first model as slightly better, while some of them were inconclusive; however, in the big picture the second model achieved a slightly better fit for all sample sizes. Moreover, the value of the correlation parameter in the first model was estimated as close to zero.

Thus, the best solution to the problem of testing the compensation account is to express directly the true (measurement error free) variables in the fitted model (either SEM or graphical model) and test the relationship between them. Acceptable, albeit much less trustworthy, solutions are either to use an alternative measure of pretest/posttest instead of the primary one in the correlation test, or to include a control group in the study and to compare the possible compensation effect between the control and the experimental (trained/stimulated) group. These methods can be used with the restriction that only a positive outcome of these tests is credible because a negative outcome can show that there is insufficient test power, or that the effect is nonexistent. Finally, neither a simple linear model test nor a naïve correlation should ever be used to test the compensation account!

## Discussion

This study aimed to methodologically assess the existing and potential evidence in favor of the popular compensation account of cognitive training and neuronal stimulation; it predicts the training effect size to be negatively related to the baseline performance tested before the training. However, most such evidence consists of negative Pearson correlations of pretest score and training gain, the latter expressed as the difference between post-test and pretest. A relatively simple mathematic derivation demonstrated that such an outcome occurs naturally when gain (treated as the dependent variable) is the linear function of the independent variable (pretest); that is a specific case of a more general statistical artifact called regression to the mean. This conclusion was supported by numerical simulations, showing a robust tendency towards reporting negative correlations even when the pretest and posttest scores are in fact unrelated. As a result, one must conclude that most of the existing evidence in favor of the compensation account is questionable (e.g., Chan et al., 2015; Cox, 1994; Dahlin, 2011; Gaultney et al., 1996; Karbach et al., 2015; Zinke et al., 2012, 2014).

Furthermore, using numerical simulations we examined if the four alternative methods (correlation with an alternative pretest measure, simple linear regression model, linear regression model including control group, and structural equation model) can validly evaluate the magnitude of the compensation effect when it is present in data, as well as validly report its lack when it is absent. Both the linear regression model and naïve correlation yielded false alarms, detecting compensation whether or not it was present in data. On the other hand, correlation with an alternative measure failed to detect a true compensation effect in a small sample. Also, including a control group and examining the interaction between group and pretest value did not lead to correct discrimination between spurious and real compensation. However, similarly to correlation with another measure, the low power of the tests was probably the reason that the effect was missed. The only fully valid detection of compensation was achieved by the use of an SEM, which diagnosed properly in both datasets and for all sample sizes.

However, the present study should not be interpreted as an argument against the compensation account, as the account itself might be valid. Simply, the empirical status of this hypothesis is still indeterminate as the validity of the methods used to corroborate it is doubtful. With proper methods, the account may in principle be supported by future data. Consequently, the merit of the present study lies in stimulating methodologically valid research on the individual differences in training and stimulation effects. Knowledge on such differences is very important because it informs who should be primarily targeted by increasingly common but costly cognitive training programs (Román et al., 2016; Schmiedek, Lövdén, & Lindenberger, 2010) or transcranial stimulation (Jaušovec & Pahor, 2017; Santarnecchi et al., 2015), which might help various subpopulations to improve performance. In fact, some studies (e.g., Au et al., 2015; Santarnecchi et al., 2016) that used methods beyond naïve correlation indeed suggested that people whose performance is worse at baseline may especially benefit from such programs. However, the only two studies that validly applied an SEM (Guye et al., 2017; Lövdén et al., 2012) provided results that support both the compensation account and the magnification account; this suggests that both compensation and magnification can occur, depending on the faculty trained and the procedures applied (see Borella, Carbone, Pastore, Beni, & Carretti, 2017). More reliable future studies are definitely needed before any firmer conclusions can be drawn. The most important take-home message from the present analysis is that such studies need reliable statistical methods.

## Appendix

**Table 7:** Comparison of the goodness-of-fit measures of the two structural equation models for the two datasets

	Dataset 1 (real compensation)			Dataset 2 (regression to the mean)		
	Model 1	Model 2	Relative fit	Model 1	Model 2	Relative fit
*N* = 28						
*χ*^2^[*D**F*]	1.9 [2]	6.25 [3]	4.05 [1]	1.9 [2]	3 [3]	0.89 [1]
*p*(*χ*^2^)	.39	.1	.044 *	.39	.39	.34
*R**M**S**E**A*[95*%**C**I*]	.057 [.0004,.338]	.167 [.034,.4]	—	.057 [.0003,.338]	.057 [.0004,.296]	—
*CFI*	.985	.908	—	.99	.986	—
*AIC*	322	325	—	332	332	—
*BIC*	338	339	—	348	346	—
*ρ*[95*%**C**I*]	− .58[− 1,−.074]	—	—	.057 [−.72,.83]	—	—
*p*(*ρ*)	.11	—	—	.5	—	—
*N* = 48						
*χ*^2^[*D**F*]	1.82 [2]	8.41 [3]	6.33 [1]	1.87 [2]	2.92 [3]	0.85 [1]
*p*(*χ*^2^)	.4	.04 *	.012 *	.39	.4	.36
*R**M**S**E**A*[95*%**C**I*]	.04 [.0001,.255]	.174 [.052,.334]	—	.042 [.0002,.257]	.042 [.0002,.224]	—
*CFI*	.992	.916	—	.994	.992	—
*AIC*	545	550	—	562	561	—
*BIC*	567	570	—	585	582	—
*ρ*[95*%**C**I*]	− .57[−.9,−.22]	—	—	.034 [−.52,.57]	—	—
*p*(*ρ*)	.04 *	—	—	.51	—	—
*N* = 80						
*χ*^2^[*D**F*]	1.84 [2]	12.32 [3]	10 [1]	1.84 [2]	2.84 [3]	1.04 [1]
*p*(*χ*^2^)	.4	.007 **	.0016 **	.4	.42	.37
*R**M**S**E**A*[95*%**C**I*]	.032 [.0001,.198]	.185 [.079,.308]	—	.032 [.0001,.198]	.031 [0,.17]	—
*CFI*	.995	.917	—	.997	.996	—
*AIC*	901	909	—	929	928	—
*BIC*	929	935	—	958	954	—
*ρ*[95*%**C**I*]	− .57[−.82,−.31]	—	—	.018 [−.39,.42]	—	—
*p*(*ρ*)	.004 **	—	—	.51	—	—
*N* = 300						
*χ*^2^[*D**F*]	1.8 [2]	38.2 [3]	36.17 [1]	1.77 [2]	2.78 [3]	0.81 [1]
*p*(*χ*^2^)	.41	< .001 ***	< .001 ***	.41	.42	.37
*R**M**S**E**A*[95*%**C**I*]	.016 [0,.101]	.196 [.143,.254]	—	.016 [0,.101]	.015 [0,.087]	—
*CFI*	.999	.915	—	.999	.999	—
*AIC*	334	338	—	345	345	—
*BIC*	339	342	—	349	349	—
*ρ*[95*%**C**I*]	− .57[−.69,−.44]	—	—	.0045 [−.19,.2]	—	—
*p*(*ρ*)	< .001 ***	—	—	.5	—	—

**p* < .05, ** *p* < .01, *** *p* < .001

**Table 8:** Estimation of linear regression models parameters (dataset 3)

Predictor	*B*	95%CI	*p*
*N* = 28			
(Intercept)	0.8	[0.19, 1.41]	.01*
Group	− 0.006	[− 0.87, 0.85]	.988
Pretest	0.67	[0.15, 1.19]	.01*
Group × pretest	− 0.27	[− 1.02, 0.48]	.45
*N* = 48			
(Intercept)	0.8	[0.36, 1.25]	.001 **
Group	− 0.0033	[− 0.64, 0.63]	.991
Pretest	0.67	[0.3, 1.05]	.001 **
Group × pretest	− 0.27	[− 0.8, 0.27]	.31
*N* = 80			
(Intercept)	0.8	[0.46, 1.13]	< .001 ***
Group	0.0042	[− 0.48, 0.48]	.99
Pretest	0.67	[0.38, 0.95]	< .001 ***
Group × pretest	− 0.27	[− 0.67, 0.14]	.19
*N* = 300			
(Intercept)	0.8	[0.63, 0.97]	< .001 ***
Group	0	[− 0.24, 0.24]	.99
Pretest	0.67	[0.53, 0.81]	< .001 ***
Group × pretest	− 0.27	[− 0.47,− 0.07]	.0088 **

The dependent variable is the posttest score

**p* < .05, ** *p* < .01, *** *p* < .001
